# Musculoskeletal injuries in athletes from five modalities: a cross-sectional study

**DOI:** 10.1186/s12891-020-3141-8

**Published:** 2020-02-24

**Authors:** Rodrigo Araújo Goes, Lucas Rafael Lopes, Victor Rodrigues Amaral Cossich, Vitor Almeida Ribeiro de Miranda, Olívia Nogueira Coelho, Ricardo do Carmo Bastos, Letícia Aparecida Marincolo Domenis, João Antonio Matheus Guimarães, João Alves Grangeiro-Neto, Jamila Alessandra Perini

**Affiliations:** 1 Centro de Trauma do Esporte, Instituto Nacional de Traumatologia e Ortopedia (INTO), Rio de Janeiro, Brazil; 2 Research Division, Instituto Nacional de Traumatologia e Ortopedia, Avenida Brasil, 500, Rio de Janeiro, RJ 20940-070 Brazil; 3 Laboratório de Pesquisa de Ciências Farmacêuticas, Centro Universitário Estadual da Zona Oeste (UEZO), Rio de Janeiro, Brazil; 4 Programa de Pós-graduação em Saúde Pública e Meio Ambiente, Escola Nacional de Saúde Pública, Fundação Oswaldo Cruz (Fiocruz), Rio de Janeiro, Brazil; 5 Escola de Educação Física e Desportos (EEFD), Universidade Federal do Rio de Janeiro (UFRJ), Rio de Janeiro, RJ Brazil

**Keywords:** Sports injury, Epidemiology, Tendinopathy, Joint injury, Muscle injury

## Abstract

**Background:**

Musculoskeletal injuries (MSK-I) are a serious problem in sports medicine. Modifiable and non-modifiable factors are associated with susceptibility to these injuries. Thus, the aim of this study was to describe the prevalence of and identify the factors associated with MSK-I, including tendinopathy and joint and muscle injuries, in athletes.

**Methods:**

In this cross-sectional observational study, 627 athletes from rugby (*n* = 225), soccer (*n* = 172), combat sports (*n* = 86), handball (*n* = 82) and water polo (*n* = 62) were recruited at different sports training centres and competitions. Athlete profiles and the prevalence of MSK-I were assessed using a self-reported questionnaire. Only previous MSK-I with imaging confirmation and/or a positive physical exam by a specialized orthopaedist were considered. The association of the epidemiological, clinical and sports profiles of athletes with MSK-I was evaluated by a logistic regression model.

**Results:**

The mean age was 25 ± 6 years, and 60% of the athletes were male. The epidemiological, clinical and sports profiles of the athletes were different for the five sport groups. The MSK-I prevalence among all athletes was 76%, with 55% of MSK-I occurring in a joint, 48% occurring in a muscle and 30% being tendinopathy, and 19% of athletes had three investigated injuries. The MSK-I prevalence and injury locations were significantly different among sport groups. There was a predominance of joint injury in combat sports athletes (77%), muscle injury in handball athletes (67%) and tendinopathy in water polo athletes (52%). Age (≥30 years) was positively associated with joint (OR = 5.2 and 95% CI = 2.6–10.7) and muscle (OR = 4.9 and 95% CI = 2.4–10.1) injuries and tendinopathy (OR = 4.1 and 95% CI = 1.9–9.3).

**Conclusion:**

There is a high prevalence of tendinopathy and joint and muscle injuries among rugby, soccer, combat sports, handball and water polo athletes. The analysis of associated factors (epidemiological, clinical and sports profiles) and the presence of MSK-I in athletes suggests an approximately 4–5-fold increased risk for athletes ≥30 years of age. The identification of modifiable and non-modifiable factors can contribute to implementing surveillance programmes for MSK-I prevention.

## Background

Musculoskeletal injuries (MSK-I) are some of the most severe health problems in sports medicine, resulting in high economic costs, withdrawal of athletes from training and competitions and potentially affecting athlete performance [1]. The prevalence of injury types and locations is different according to sport modality, varying from 5 to 60% for joint injuries [2, 3], 20–60% for muscle injuries [4, 5] and 10–50% for tendinopathy [6].

Non-modifiable and modifiable factors have been associated with MSK-I [1]. The multifactorial and dynamic nature of the MSK-I highlights the importance of knowing the confounding variables to assist biostatistical methods of surveillance in athlete health, which may contribute to injury prevention programmes, helping professionals involved with the training of athletes [7]. Thus, understanding the interaction between epidemiological and etiological factors involved with MSK-I is essential to the development and implementation of sports injury surveillance programmes [8]. Recently, our group showed that genetic factors were associated with tendinopathy development and were able to contribute to the identification of new therapeutic targets and personalized training programmes to prevent injury in athletes [9, 10].

As far as we know, there are no studies comparing athlete profiles and their associated factors with MSK-I among different sport modalities. Thus, the aim of this study was to describe the epidemiological, clinical and athletic profile of five sport modalities to verify the prevalence of and associated factors for tendinopathies and joint and muscle injuries in athletes.

## Methods

### Population and study design

The Human Ethics Committee of the *Instituto Nacional de Traumatologia e Ortopedia Jamil Haddad* approved the study (protocol number 2.455.630/2017). A cross-sectional study was conducted with Brazilian athletes regarding the prevalence of and factors associated with MSK-I. The inclusion criteria were Brazilian athletes aged 18–45 years old who were symptomatic or asymptomatic for any MSK-I. All MSK-I diagnoses were confirmed by two blinded specialized orthopaedists. Athletes with a history of MSK-I for reasons unrelated to sports practice were excluded from the present study. Six hundred and twenty-seven athletes from the following sports were recruited between March and December 2018 at different sports training centres and competitions: 225 rugby, 172 soccer, 86 combat sports, 82 handball and 62 water polo. The participants provided written informed consent and answered a questionnaire about their demographic, epidemiological, clinical and sports profiles. All questionnaires were checked by an expert researcher together with the athlete.

### Questionnaire

Brazilian athletes’ profiles and MSK-I history were assessed using a self-reported questionnaire previously validated by an expert panel, which was divided into three sections regarding general, training and MSK-I-specific information (Additional file 1). First, the athletes reported general information that about their sociodemographic characteristics such as age, sex, skin colour, level of schooling (middle school, high school or university education), family income, and anthropometric measures (height and body mass index - BMI). Skin colour was reported according to the classification scheme of the Brazilian official census (*Instituto Brasileiro de Geografia e Estatística –* IBGE), which employs only a few pre-established colour categories based on self-classification: white, intermediate, black, yellow or indigenous [11]. Family income also was categorized according to IBGE: A > 20, B = 10 to 20, C = 5 to 10 or D = 2 to 5 minimum wages. In addition, clinical characteristics such as nutritional monitoring (during sports careers), smoking (cigarette, hand-rolled tobacco, pipe, cigar or hookah), and alcohol consumption were also included as general information. Smoking was assessed as the number of times per day [12]. Alcohol consumption was assessed by the frequency of consumption per week (low: < 7 doses/week, moderate: 7 to 12 doses/week or high: > 21 doses/week) [13]. However, in this study, the athletes were categorized as “Yes” or “No” for smoking or drinking alcohol, regardless of the types and frequencies. The second section of the questionnaire was about sports and training characteristics. The athletes detailed the sport modality, their age at the beginning of competitive practice, the years of training and the weekly training hours. Finally, the third section of the questionnaire was regarding the MSK-I. The athletes detailed the type, location, number of episodes and time withdrawn from sports activities for injuries, according to Fuller and colleagues [14]. Of all self-reported injuries, only those with previous positive diagnoses by two blinded specialized orthopaedists (physical exam and/or imaging) were considered for this study.

### Prevalence of MSK-I

The athletes reported a history of MSK-I and described the specific sites concerning muscle injuries (thorax, shoulder, arm and forearm, hip, thigh [anterior/posterior], leg [lateral/medial], calf, or others), joint injury or tendinopathy (shoulder, elbow, hand, hip, knee, ankle or others). The prevalence of MSK-I was calculated as the total number of injured athletes divided by the total number of athletes in each selected sport group, according to the self-reported questionnaire and a positive physical exam or imaging exam.

### Statistical analysis

The normally distribution of studied population was determined by the Shapiro-Wilk test. Continuous variables were reported as mean ± standard deviation (SD), differences in these values between sports modalities were tested by one-way analysis of variance (ANOVA). However, according to their distribution and clinical significance, for the analysis, continuous variables (age, height and age at the beginning of sport practice) were divided into quartiles, while years of training and weekly training hours were categorized into tertiles. Categorical data were shown in proportions and differences among sports using the Chi-squared (χ2) statistic test or Fischer exact test, when applicable.

Multivariable logistic regression analyses were performed to identify possible confounding factors in the associations between sociodemographic, clinical, and athletic characteristics and joint and muscle injury or tendinopathy, which was estimated by the odds ratio (OR) with a 95% confidence interval (95% CI). Univariate characteristics with a *p*-value less than 0.25 were included in the multivariable logistic regression analysis. The difference was statistically significant when *p*-value was less than or equal to 0.05. All analyses were performed using the IBM SPSS 20.0 Statistics for Windows (SPSS Inc., Chicago, IL, USA).

## Results

**Fig. 1:**
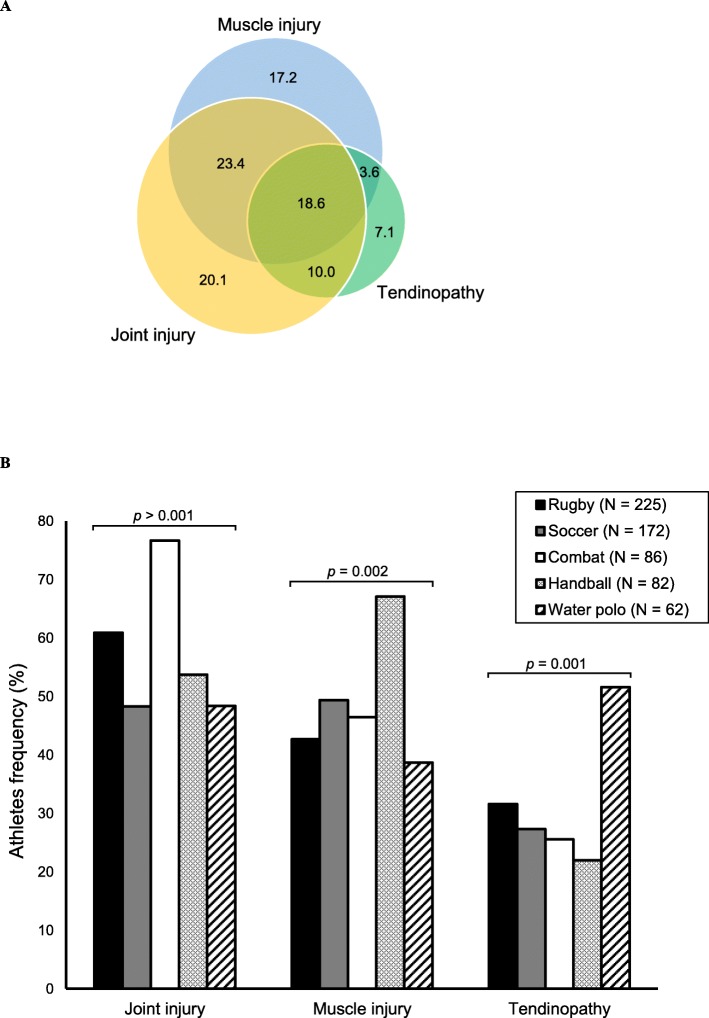
Distribution of musculoskeletal injuries in athletes (*n* = 478). **a** Venn diagram of injuries. **b** Frequency of the musculoskeletal injuries in athletes by sports group. *P*-value ≤0.05 was obtained through the Chi-squared Test (Pearson *p*-value)

The athletes’ mean age was 24.7 ± 5.7 years old, 374 (59.6%) were male, 334 (53.3%) had a university education, and the mean BMI was 24.8 ± 3.5 kg/m^2^. Four hundred and seventy-eight athletes (76.2%) reported MSK-I, and 89 (18.6%) athletes presented a history of multiple injuries (Fig. 1a). The prevalence of injuries in all recruited athletes was 55.0% (*n* = 345) for joint injuries, 47.8% (*n* = 300) for muscle injuries and 30.3% (*n* = 190) for tendinopathies. The Fig. 1b shows the prevalence of MSK-I in athletes by sport group. Sociodemographic, clinical and athletic characteristics categorized by sport groups, in addition to injury types and locations, are described in the Table 1.

**Table 1:** Socio-demographic, clinical, sport, training characteristics and injuries locations by sport group

Variables	Rugby *n* = 225	Soccer *n* = 172	Combat n = 86	Handball *n* = 82	Water polo *n* = 62	*P-*value^a^
			n (%)			
Age (years)						
< 20	28 (12.4)	58 (33.7)	15 (17.4)	12 (14.6)	20 (32.3)	< 0.001
20 to 24	96 (42.8)	47 (27.3)	32 (37.2)	27 (32.9)	20 (32.3)	
25 to 29	64 (28.4)	33 (19.2)	22 (25.6)	22 (26.8)	12 (19.3)	
≥ 30	37 (16.4)	34 (19.8)	17 (19.8)	21 (25.7)	10 (16.1)	
Sex						
Female	160 (71.1)	0 (0.0)	19 (22.1)	48 (58.5)	26 (41.9)	< 0.001
Male	65 (28.9)	172 (100.0)	67 (77.9)	34 (41.5)	36 (58.1)	
Height (centimeters)						
< 165	89 (39.6)	5 (2.9)	16 (18.6)	7 (8.5)	9 (14.5)	< 0.001
165 to 174	72 (32.0)	45 (26.2)	36 (41.9)	35 (42.7)	17 (27.4)	
175 to 184	49 (21.8)	72 (41.9)	25 (29.0)	21 (25.6)	15 (24.2)	
≥ 185	15 (6.6)	50 (29.1)	9 (10.5)	19 (23.2)	21 (33.9)	
Alcohol consumption^b^						
No	66 (29.3)	95 (55.2)	50 (58.8)	32 (40.0)	15 (24.2)	< 0.001
Yes	159 (70.7)	77 (44.8)	35 (41.2)	48 (60.0)	47 (75.8)	
Smoking^c^						
No	198 (88.0)	166 (96.5)	85 (98.8)	77 (95.1)	59 (95.2)	0.002
Yes	27 (12.0)	6 (3.5)	1 (1.2)	4 (4.9)	3 (4.8)	
Age at the beginning of sport practice (years)						
0 to 8	3 (1.3)	109 (63.3)	35 (40.7)	8 (9.8)	9 (14.5)	< 0.001
9 to 13	23 (10.2)	43 (25.0)	23 (26.8)	43 (52.4)	34 (54.9)	
14 to 18	84 (37.4)	20 (11.6)	21 (24.4)	27 (32.9)	19 (30.6)	
> 18	115 (51.1)	0 (0.0)	7 (8.1)	4 (4.9)	0 (0.0)	
Years of training						
0 to 5	131 (58.2)	8 (4.7)	9 (10.5)	12 (14.7)	8 (12.9)	< 0.001
6 to 10	71 (31.6)	44 (25.5)	19 (22.1)	22 (26.8)	29 (46.8)	
> 10	23 (10.2)	120 (69.8)	58 (67.4)	48 (58.5)	25 (40.3)	
Weekly training hours						
0 to 7	87 (38.7)	23 (13.4)	15 (17.4)	23 (28.1)	9 (14.5)	< 0.001
8 to 14	104 (46.2)	83 (48.3)	16 (18.6)	38 (46.3)	13 (21.0)	
15 to 21	25 (11.1)	44 (25.6)	33 (38.4)	17 (20.7)	30 (48.4)	
> 21	9 (4.0)	22 (12.8)	22 (25.6)	4 (4.9)	10 (16.1)	
Joint injury	233 (100.0)	100 (100.0)	158 (100.0)	36 (100.0)	51 (100.0)	< 0.001
Hand	43 (18.4)	3 (3.0)	24 (15.2)	1 (2.8)	8 (15.7)	
Elbow	11 (4.7)	0 (0.0)	22 (13.9)	4 (11.1)	8 (15.7)	
Shoulder	58 (24.9)	15 (15.0)	34 (21.5)	7 (19.5)	16 (31.4)	
Knee	44 (18.9)	45 (45.0)	45 (28.5)	11 (30.5)	12 (23.5)	
Ankle	72 (30.9)	34 (34.0)	27 (17.1)	12 (33.3)	3 (5.9)	
Hip	5 (2.2)	3 (3.0)	6 (3.8)	1 (2.8)	4 (7.8)	
Muscle injury	192 (100.0)	115 (100.0)	87 (100.0)	101 (100.0)	43 (100.0)	< 0.001
Thoracic	12 (6.2)	1 (0.9)	8 (9.2)	3 (3.0)	2 (4.6)	
Forearm and arm	17 (8.8)	1 (0.9)	8 (9.2)	9 (8.9)	8 (18.7)	
Shoulder	33 (17.2)	3 (2.6)	19 (21.8)	16 (15.8)	20 (46.5)	
Anterior thigh	27 (14.1)	41 (35.7)	10 (11.5)	16 (15.8)	2 (4.6)	
Posterior thigh	42 (21.9)	48 (41.7)	15 (17.3)	28 (27.8)	2 (4.6)	
Lateral/medial leg	18 (9.4)	2 (1.7)	6 (6.9)	11 (10.9)	0 (0.0)	
Calf	26 (13.5)	15 (13.0)	10 (11.5)	7 (6.9)	2 (4.6)	
Hip	14 (7.3)	3 (2.6)	7 (8.0)	5 (5.0)	7 (16.4)	
Others	3 (1.6)	1 (0.9)	4 (4.6)	6 (5.9)	0 (0.0)	
Tendinopathy	84 (100.0)	51 (100.0)	30 (100.0)	21 (100.0)	38 (100.0)	< 0.001
Hand	9 (10.7)	3 (5.9)	9 (30.0)	2 (9.5)	1 (2.6)	
Elbow	0 (0.0)	0 (0.0)	3 (10.0)	2 (9.5)	7 (18.4)	
Shoulder	23 (27.4)	9 (17.6)	9 (30.0)	7 (33.3)	25 (65.8)	
Knee	30 (35.7)	30 (58.9)	3 (10.0)	9 (42.9)	4 (10.6)	
Ankle	18 (21.4)	9 (17.6)	4 (13.3)	0 (0.0)	1 (2.6)	
Others	4 (4.8)	0 (0.0)	2 (6.7)	1 (4.8)	0 (0.0)	

^a^*P*-value ≤0.05 was obtained through the Chi-squared Test (Pearson p-value). ^b^ Information were obtained from 85 combat athletes and 80 handball athletes. ^c^ Information were obtained 81 handball athletes

Detailed descriptions of athletes’ sociodemographic, clinical and athletic characteristics are shown by sport group.

### Rugby

Rugby athletes (age: 24.5 ± 4.6 years old, height: 170 ± 10 cm, BMI: 24.9 ± 4.3 kg/m^2^) were mostly self-declared as having white skin colour (54.7%, *n* = 123), and 66.7% (*n* = 150) had a university education, 88 (39.1%) D class family income and 86 (38.2%) nutritional follow-up. Regarding the training time of athletes, the mean age at the beginning of sport practice was 18.6 ± 4.7 years, with the years of practice in the sport being 5.6 ± 4.4 years and the training time being 9.9 ± 5.7 h/week. Of the 225 athletes, 170 (75.6%) reported a history of MSK-I. The most frequent injury types were joint (*n* = 137, 60.9%) and muscle (*n* = 96, 42.7%) (Fig. 1b). Five hundred and nine injuries were identified in total, of which the shoulder and lower extremities were the most affected locations (Table 1).

### Soccer

Soccer athletes (age: 24.6 ± 6.9 years old, height: 180 ± 20 cm, BMI: 23.6 ± 3.7 kg/m^2^) were mostly self-declared as intermediate (35.5%, *n* = 61) and white skin colour (33.7%, *n* = 58), and 117 (68.0%) had a high school degree, 62 (36.0%) C class family income and 115 (66.9%) nutritional monitoring. The training exposure of athletes showed that the mean age at the beginning of sport practice was 8.4 ± 3.5 years, with more practice time in the sport (13.9 ± 6.1 years) and weekly training hours (14.0 ± 7.0 h). One hundred thirty-four (77.9%) athletes had a history of MSK-I. Muscle and joint injuries were the most reported injuries in this sport (*n* = 85, 49.4% and *n* = 83, 48.3, respectively) (Fig. 1b). The main locations of muscle injury were the posterior and anterior thigh muscles (41.7 and 35.7%, respectively), while joint injury and tendinopathy were more frequent in the knee (Table 1).

### Combat sports

The combat sports group comprised athletes from judo (*n* = 48, 55.8%), Brazilian jiu-jitsu (*n* = 18, 20.9%), kickboxing (*n* = 8, 9.4%), MMA (*n* = 7, 8.1%) and wrestling (n = 5, 5.8%), with an age mean of 25.5 ± 6.6 years, height of 170 ± 10 cm and BMI of 25.8 ± 4.1 kg/m^2^. The majority of athletes self-declared an intermediate skin colour (*n* = 34, 39.5%), had a university education (*n* = 54, 62.8%), had C or D class family income (*n* = 70, 81.4%) and had nutritional follow-up (*n* = 68, 79.1%). The mean age at the beginning of sport practice was 10.7 ± 5.9 years; thus, the training time of the athletes was 14.0 ± 6.9 years, with a mean weekly training duration of 16.1 ± 7.4 h. Among the reported MSK-I, joint injury was most prevalent (*n* = 66, 76.7%) in athletes (Fig. 1b), mainly in the knee and shoulder (Table 1).

### Handball

The mean age of handball athletes was 25.2 ± 5.3 years, the mean height was 180 ± 10 cm, and the mean BMI was 24.1 ± 3.6 kg/m^2^. Forty-eight athletes reported skin colour according to self-perception, of which 23 (47.9%) athletes were self-classified as intermediate. In addition, 53 (64.6%) had a university education, 44 (53.7%) had D class family income, and 31 (37.8%) had nutritional monitoring. The athletes reported that they started practicing the sport approximately 12.8 ± 4.0 years old; thus, training exposure characteristics showed that the mean time for participating in the sport was 11.9 ± 5.4 years, and the training time was 10.9 ± 5.7 h/week. Muscle injury was more prevalent (*n* = 55, 67.1%) in this sport modality (Fig. 1b). The most affected locations were the ankle and knee for joint injuries, the posterior thigh for muscle injuries and the knee and shoulder for tendinopathy (Table 1).

### Water polo

Athletes (age: 23.4 ± 5.1 years old, height: 180 ± 10 cm, BMI: 25.4 ± 3.7 kg/m^2^) were mostly self-declared as having white skin colour (59.7%, *n* = 37), 38 (61.3%) had a university education, 52 (83.9%) had B and D class family income, and 36 (58.1%) performed nutritional follow-up. The training exposure characteristics show that these athletes started participating in sports approximately 11.8 ± 2.7 years old, had 11.5 ± 6.1 years of practice in the sport and approximately 15.3 ± 7.3 training hours/week. Thirty-two (51.6%) athletes reported a history of tendinopathy; the shoulder (65.8%) was the most affected site (Table 1) and water polo sport with a higher prevalence of this injury (Fig. 1b).

The Table 2 shows the multivariate logistic regression model used to identify the factors associated with tendinopathy and joint and muscle injuries regardless of sport group.

**Table 2:** Associated factors with joint injury, muscle injury and tendinopathy from logistic regression model

Variables	Control *n* = 141	Joint injury *n* = 345	OR adjusted^a^ (CI 95%)	Muscle injury *n* = 300	OR adjusted^b^ (CI 95%)	Tendinopathy *n* = 190	OR adjusted^c^ (CI 95%)
	n (%)			n (%)		n (%)	
Age (years)							
< 20	50 (35.5)	48 (13.9)	1^d^	47 (15.7)	1^d^	28 (14.7)	1^d^
20 to 24	52 (36.9)	120 (34.8)	2.39 (1.42–4.02)	91 (30.3)	1.95 (1.14–3.33)	61 (32.1)	2.11 (1.14–3.92)
25 to 29	24 (17.0)	98 (28.4)	4.08 (2.21–7.51)	83 (27.7)	3.60 (1.94–6.69)	57 (30.0)	4.02 (2.02–8.01)
≥ 30	15 (10.6)	79 (22.9)	5.22 (2.55–10.67)	79 (26.3)	4.95 (2.42–10.09)	44 (23.2)	4.14 (1.85–9.26)
Sex							
Female	57 (40.4)	147 (42.6)	1^d^	118 (39.3)	1^d^	82 (43.2)	1^d^
Male	84 (59.6)	198 (57.4)	0.98 (0.62–1.53)	182 (60.7)	1.02 (0.64–1.63)	108 (56.8)	0.80 (0.47–1.35)
Height (centimeters)							
< 165	33 (23.4)	75 (21.7)	1^d^	51 (17.0)	1^d^	36 (19.0)	1^d^
165 to 174	45 (31.9)	118 (34.2)	1.12 (0.63–2.02)	91 (30.3)	1.13 (0.60–2.11)	66 (34.7)	1.08 (0.55–2.13)
175 to 184	34 (24.1)	99 (28.7)	1.28 (0.70–2.34)	103 (34.3)	1.78 (0.93–3.34)	57 (30.0)	1.29 (0.64–2.60)
≥ 185	29 (20.6)	53 (15.4)	0.84 (0.43–1.63)	55 (18.4)	1.19 (0.59–2.41)	31 (16.3)	0.79 (0.37–1.73)
Alcohol consumption^e^							
No	60 (42.6)	137 (39.8)	1^d^	110 (37.0)	1^d^	60 (31.9)	1^d^
Yes	81 (57.4)	207 (60.2)	0.97 (0.62–1.50)	187 (63.0)	1.05 (0.68–1.65)	128 (68.1)	1.30 (0.79–2.15)
Smoking^f^							
No	134 (95.0)	317 (92.2)	1^d^	275 (92.0)	1^d^	173 (91.1)	1^d^
Yes	7 (5.0)	27 (7.8)	1.42 (0.57–3.53)	24 (8.0)	1.39 (0.54–3.56)	17 (8.9)	1.91 (0.72–5.07)
Sport group							
Rugby	55 (39.0)	137 (39.7)	1^d^	96 (32.0)	1^d^	71 (37.4)	1^d^
Soccer	38 (27.0)	83 (24.1)	0.70 (0.36–1.39)	85 (28.3)	1.03 (0.52–2.05)	47 (24.7)	0.70 (0.31–1.60)
Combat	14 (9.9)	66 (19.1)	1.33 (0.60–2.93)	40 (13.4)	1.18 (0.50–2.76)	22 (11.6)	0.74 (0.28–1.95)
Handball	13 (9.2)	29 (8.4)	0.56 (0.24–1.31)	55 (18.3)	1.73 (0.78–3.82)	18 (9.5)	0.51 (0.19–1.35)
Water polo	21 (14.9)	30 (8.7)	0.45 (0.21–0.96)	24 (8.0)	0.59 (0.27–1.31)	32 (16.8)	0.77 (0.33–1.79)
Years of training							
0 to 5	47 (33.3)	85 (24.6)	1^d^	62 (20.7)	1^d^	40 (21.1)	1^d^
6 to 10	46 (32.6)	101 (29.3)	1.13 (0.65–1.96)	81 (27.0)	1.15 (0.64–2.06)	55 (28.9)	1.39 (0.72–2.67)
> 10	48 (34.1)	159 (46.1)	1.27 (0.71–2.26)	157 (52.3)	1.56 (0.87–2.81)	95 (50.0)	1.65 (0.85–3.21)
Weekly training hours							
0 to 7	42 (29.8)	88 (25.5)	1^d^	68 (22.7)	1^d^	44 (23.1)	1^d^
8 to 14	55 (39.0)	131 (38.0)	1.03 (0.60–1.76)	125 (41.6)	1.21 (0.69–2.10)	68 (35.8)	0.98 (0.53–1.83)
15 to 21	32 (22.7)	82 (23.8)	1.19 (0.63–2.24)	69 (23.0)	1.17 (0.61–2.23)	52 (27.4)	1.48 (0.73–3.00)
> 21	12 (8.5)	44 (12.7)	1.59 (0.71–3.60)	38 (12.7)	1.63 (0.72–3.70)	26 (13.7)	1.67 (0.68–4.10)

*OR* is Odds ratio, *CI* is confidence interval. ^a^OR adjusted by Age, Sport group, Years of training and Weekly training hours. ^b^OR adjusted by Age, Height, Smoking, Sport group and Years of training. ^c^OR adjusted by Age, Drinking alcohol, Smoking, Sport group, Years of training and Weekly training hours. ^d^Reference value. ^e^Information were obtained from 344 athletes from joint injury group, 297 athletes from muscle injury group and 188 athletes from tendinopathy group. ^f^Information were obtained from 344 athletes from joint injury group and 299 athletes from muscle injury group

## Discussion

High prevalence of and risks associated with MSK-I have been gaining attention in sports medicine due to the negative impact on health and athletic performance [8]. Some sports leagues have proposed a definition for MSK-I that as a medically diagnosed physical complaint sustained while undertaking competition or training that has been accepted by some sports unions [14, 15]. In the present study, there was a high prevalence of MSK-I (76%) among Brazilian rugby, soccer, combat, handball and water polo athletes, and 19% reported a history of tendinopathy and joint and muscle injuries in combination. Our result is in agreement with that of Graças and colleagues, who observed a prevalence of 82.6% of MSK-I in Brazilian jiu-jitsu athletes [16]. In addition, 65% of first division soccer athletes showed some MSK-I type during the season [17], and 58% of university athletes from fifteen different sports had at least one injury at the end of the sports season [18]. The accumulation of MSK-I makes athletes less tolerant of hard training and therefore less likely to achieve higher goals, in addition to the serious problems they present for sports medical teams [19]. MSK-Is also generate high economic costs due to performance loss and diagnosis and treatment of the injuries, affecting the athlete and the sports team [20].

The prevalence/incidence of types and locations, causes, and characteristics of MSK-Is varies according to sport modality [8, 21, 22]. In the present study, there were significant differences in the prevalence of the type and location of tendinopathy and joint and muscle injuries according to sport groups. Rugby involves a high volume of running and cutting and turning movements with high speed. It also involves serious direct trauma causing severe joint injuries in the ankle, shoulder and knee muscles or tendons [19, 23]. Due to this, in the present study, we observed a high frequency of injury in the ankles and shoulders of rugby athletes. Soccer is a team sport with intense movements such as cutting, jumping, fast running and ball kicking [20, 24]. Dönmez and colleagues observed that posterior thigh muscle fatigue and patellar tendon overuse were the most common injuries in Turkish soccer athletes [21], which corroborates the high frequency of posterior thigh muscle injuries and knee tendinopathy in the present study. Combat sports involve direct body contact with an opponent through a strike, kick and/or throw, which is reflected in the high frequency of joint injuries. For instance, judo and wrestling athletes are more likely to incur upper limb injuries due to the blows of pulls and holding the opponent, while taekwondo athletes are more susceptible to lower limb injuries because of kicks [8, 25]. In the present study, joint injury was more prevalent, mainly in the knee and shoulder, in combat athletes. Handball is a high-intensity sport, involving interaction with opponents and performing different intense body movements, such as overhead throwing [26, 27]. The prevalence of injuries in the ankle and knee joint and posterior thigh muscle found in our study can be explained by the dynamics of spin acceleration and jumping and landing tasks with only one foot [26, 28]. Water polo is a team and contact sport with dynamic movements without the contribution of a solid base of support, which may cause micro-tears of the musculoskeletal structures, mainly the shoulder tendon, due to repetitive throwing movements [29–31]. In the present study, the prevalence of shoulder tendinopathy was in agreement with that observed by Hams and colleagues in Australian water polo athletes [29].

According to sports modality, multiple factors can be associated with the prevalence of injuries in athletes [1, 8, 22]. Despite the differences in dynamics, logic, mechanisms, goals and training style of various sports, studies have been shown non-modifiable and modifiable risks associated with the prevalence of types and locations of MSK-I in athletes [1, 23, 30]. In the present study, only advanced age was a non-modifiable factor associated with MSK-I, regardless of the sport group. Self-reported musculoskeletal injury prevalence, depending on the study design factors and the age of the study population, varies from 2 to 65%. Age-adjusted logistic regression analyses have shown that athletes who train more than 2 h per day are 2 to 3.5 times more likely to develop an MSK-I, mainly in sports involving overuse or repetitive movements [32]. In addition, this result is in consonance with a Brazilian study, which observed a higher occurrence of MSK-I in jiu-jitsu athletes aged 30 years or more [16], and with the study of Snodgrass and colleagues, in which age was associated with muscle-tendon injury history in the neck in Australian rugby union players [33]. This can support further analytical studies by creating surveillance programmes to increase the career duration of athletes.

The risk of recall bias in self-report questionnaires and the lack of information regarding life habits are limitations of our study. In addition, this is a cross-sectional study and does not allow us to distinguish whether the exposure came before or after the observed outcome. However, a strength of our study is the relevant sample size and quality of questionnaire answers due to the high frequency of university education among the athletes. In addition, at the end of data collection, a trained observer checked the questionnaire with each athlete, and the database was double-checked by different trained researchers.

## Conclusion

There is a high prevalence of tendinopathy and joint and muscle injuries among Brazilian rugby, soccer, combat sports, handball and water polo athletes. The MSK-I (type and site) prevalence was significantly different among the five sports groups. Older age was associated with tendinopathy and joint and muscle injuries regardless of the sport group, with an approximate 4–5-fold increased risk for athletes ≥30 years of age. Modifiable or non-modifiable associated factors can encouraging analytical studies on injury surveillance with evident information on the type, location and severity of injuries among sports. Together, this information can contribute to implementing surveillance programmes to prevent MSK-Is.

## Supplementary information

**Additional file 1: Questionnaire.** Musculoskeletal injuries report for Brazilian athletes.

## Data Availability

The datasets used and/or analysed during the current study are available from the corresponding author on reasonable request.

## Abbreviations

95% CI: 95% Confidence interval
χ2: Chi-square
BMI: Body mass index
IBGE: *Instituto Brasileiro de Geografia e Estatística*
MSK-I: Musculoskeletal injuries
OR: Odds ratios
SD: Standard deviation
