# Supplementary material for: Musculoskeletal injuries in athletes from five modalities: a cross-sectional study
Source: BMC Musculoskelet Disord. 2020 Feb 24;21:122. doi: 10.1186/s12891-020-3141-8 (PMC7041260; doi:10.1186/s12891-020-3141-8)
Supplement: Supplementary file 1 — Additional file 1: Questionnaire. Musculoskeletal injuries report for Brazilian athletes. [file 12891_2020_3141_MOESM1_ESM.pdf]

QUESTIONNAIRE: **Musculoskeletal injuries report for Brazilian athletes.**

Name: ____.

Sports training centres or competitions: ____.

Phone and e-mail: ____.

Date: ____/____/_______.

Part 1 - General information:

1. Sex: ( ) Female ( ) Male **Date of birth:** ____/____/_______.

2. Weight: _______________kg. **Height:** ___________________cm.

3. Level of schooling: ( ) Middle school. ( ) High school. ( ) University education.

4. Self-classification skin colour (according to the classification scheme of the Brazilian official census – IBGE):

( ) White. ( ) Intermediate. ( ) Black. ( ) Yellow. ( ) Indigenous.

5. Family income (according to the classification scheme of the Brazilian official census - IBGE):

( ) A. ( ) B. ( ) C. ( ) D.

6. Do you have professional nutritional monitoring due to your sport career?

( ) No. ( ) Yes.

7. Do you consume alcohol? ( ) No. *Skip to question 8.* ( ) Yes.

If yes, what is the frequency of alcohol consumption?

( ) Low (< 7 doses/week). ( ) Moderate (7 to 12 doses/week). ( ) High (high: > 21 doses/week).

8. Do you smoke? ( ) No. *Skip to question 9.* ( ) Yes.

If yes, what are the type and frequency of smoking?

	Type	Frequency (number of times per day)			
( )	Cigarette	( ) ≤ 10	( ) 11 to 20	( ) 21 to 30	( ) > 30
( )	Hand-rolled	( ) ≤ 10	( ) 11 to 20	( ) 21 to 30	( ) > 30
( )	Pipe	( ) ≤ 10	( ) 11 to 20	( ) 21 to 30	( ) > 30
( )	Cigar	( ) ≤ 10	( ) 11 to 20	( ) 21 to 30	( ) > 30
( )	Hookah	( ) ≤ 10	( ) 11 to 20	( ) 21 to 30	( ) > 30

Part 2 - Sports and training information:

9. Sport modality: ____.

10. Side of dominance (for sports): ( ) Left. ( ) Right. ( ) Bilateral.

11. Coach: ( ) Physical educator/ Certified athletic trainer. ( ) Former professional athlete.
( ) Both. ( ) Other (please specify): ______________________________.

12. Age at the beginning of sport practice: __________ years old.

13. Years of training: ______________________________ years.

14. Weekly training hours: ____________________ hours per week.

Part 3 - MSK-I-specific information:

15. Joint injury

15.1. Have you ever had any joint injury? ( ) No. *Skip to question 16.* ( ) Yes.

15.2. Did the joint(s) injury(ies) occur during:
( ) Training. ( ) Competition. ( ) Other (please specify): ____________________.

15.3. What was the cause of the joint(s) injury(ies)? ( ) Overuse. ( ) Trauma.

15.4. Was the joint(s) injury(ies) caused by contact: ( ) No. ( ) Yes.

15.5. How many episodes of joint(s) injury(ies) have you experienced?
( ) ≤ 3. ( ) 4 to 9. ( ) 10 to 15. ( ) > 15.

15.6. Date of joint(s) injury(ies):____.

15.7. Joint(s) injury(ies) involved (mark all that apply):
( ) Shoulder. ( ) Elbow. ( ) Hand. ( ) Hip. ( ) Knee.
( ) Ankle. ( ) Other (please specify): ________________________________.

15.8. Side of body joint(s) injury(ies):
( ) Left. ( ) Right. ( ) Bilateral. ( ) Not applicable.

15.9. Did you withdraw from sports activities for joint(s) injury(ies)?
( ) No. *Skip to question 16.*
( ) Yes. Please specify the period ____________________________ days. *Skip to question 16.*

- **Diagnosis of joint(s) injury(ies):** (this space is to be filled up only by specialized Orthopaedists Members)

( ) Physical exam. ( ) Imaging: ____
____
____
____

Signature: ________________________ Reviewed by: ___________________________

16. Muscle injury

16.1. Have you ever had a muscle injury? ( ) No. *Skip to question 17.* ( ) Yes.

16.2. Did the muscle(s) injury(ies) occur during:

( ) Training. ( ) Competition. ( ) Other (please specify): ________________________.

16.3. What was the cause of the muscle(s) injury(ies)? ( ) Overuse. ( ) Trauma.

16.4. Was the muscle(s) injury(ies) caused by contact: ( ) No. ( ) Yes.

16.5. How many episodes of muscle(s) injury(ies) have you experienced?

( ) ≤ 3. ( ) 4 to 9. ( ) 10 to 15. ( ) > 15.

16.6. Date of muscle(s) injury(ies):____.

16.7. Muscle(s) injury(ies) involved (mark all that apply):

( ) Thorax. ( ) Shoulder. ( ) Arm. ( ) Forearm. ( ) Anterior thigh.

( ) Posterior thigh. ( ) Lateral leg. ( ) Medial leg. ( ) Calf. ( ) Hip.

( ) Other (please specify): ____.

16.8. Side of body muscle(s) injury(ies):

( ) Left. ( ) Right. ( ) Bilateral. ( ) Not applicable.

16.9. Did you withdraw from sports activities for muscle(s) injury(ies)?

( ) No. *Skip to question 17.*

( ) Yes. Please specify the period ________________________________ days. *Skip to question 17.*

- **Diagnosis of muscle(s) injury(ies):** (this space is to be filled up only by specialized Orthopaedists Members)

( ) Physical exam. ( ) Imaging: ____

____

____

____

Signature: ___________________________ Reviewed by: ______________________________

17. Tendinopathy

17.1. Have you ever had a tendinopathy? ( ) No. *The end of questionnaire.* ( ) Yes.

17.2. Did the tendinopathy(ies) occur during:

( ) Training. ( ) Competition. ( ) Other (please specify): ________________________.

17.3. What was the cause of the tendinopathy(ies)? ( ) Overuse. ( ) Trauma.

17.4. Was the tendinopathy(ies) caused by contact: ( ) No. ( ) Yes.

17.5. How many episodes of tendinopathy(ies) have you experienced?

( ) ≤ 3. ( ) 4 to 9. ( ) 10 to 15. ( ) > 15.

17.6. Date of tendinopathy(ies):____.

17.7. Injury(ies) body part involved (mark all that apply):

( ) Shoulder. ( ) Elbow. ( ) Hand. ( ) Hip. ( ) Knee.

( ) Ankle ( ) Other (please specify): ____.

17.8. Side of tendinopathy(ies):

( ) Left. ( ) Right. ( ) Bilateral. ( ) Not applicable.

17.9. Did you withdraw from sports activities for tendinopathy(ies)?

( ) No. *The end of questionnaire.*

( ) Yes. Please specify the period ______________________________ days. *The end of questionnaire.*

- **Diagnosis of tendinopathy(ies):** (this space is to be filled up only by specialized Orthopaedists Members)

( ) Physical exam. ( ) Imaging: ____

____

____

____

Signature: ______________________________ Reviewed by: _________________________________

This is the end of the questionnaire, thank you for participating.

Signature: ______________________________ Reviewed by: _________________________________

Authors: Rodrigo Araújo Goes[1], Lucas Rafael Lopes[2,3,4], Victor Rodrigues Amaral Cossich[2,5], Vitor Almeida Ribeiro de Miranda[1], Olívia Nogueira Coelho[5], Ricardo do Carmo Bastos[1], Letícia Aparecida Marincolo Domenis[1], João Antonio Matheus Guimarães[2], João Alves Grangeiro Neto[1], Jamila Alessandra Perini[2,3,4].

[1]Centro de Trauma do Esporte, Instituto Nacional de Traumatologia e Ortopedia (INTO), Rio de Janeiro, Brazil;
[2]Divisão de Pesquisa, Instituto Nacional de Traumatologia e Ortopedia (INTO), Rio de Janeiro, Brazil;
[3]Laboratório de Pesquisa de Ciências Farmacêuticas, Centro Universitário Estadual da Zona Oeste (UEZO), Rio de Janeiro, Brazil;
[4]Programa de Pós-graduação em Saúde Pública e Meio Ambiente, Escola Nacional de Saúde Pública, Fundação Oswaldo Cruz (Fiocruz), Rio de Janeiro, Brazil;
[5]Escola de Educação Física e Desportos (EEFD), Universidade Federal do Rio de Janeiro (UFRJ), Rio de Janeiro, RJ, Brazil.
